# Editorial: Sex-related differences in bone disease and musculoskeletal health

**DOI:** 10.3389/fendo.2025.1599232

**Published:** 2025-04-15

**Authors:** Giacomina Brunetti, Jonathan H. Tobias

**Affiliations:** ^1^ Department of Biosciences, Biotechnologies and Environment, University of Bari Aldo Moro, Bari, Italy; ^2^ Musculoskeletal Research Unit, Bristol Medical School, University of Bristol, Bristol, United Kingdom

**Keywords:** sex difference, sexual dimorphism, bone disease, musculoskeletal health, gender differences

Sex-related differences have been observed to influence health and disease, making an understanding of these differences a crucial step for personalized medicine. In the case of bone disease and musculoskeletal health, in addition to evident differences in bone mass and structure between the sexes, there are well-documented discrepancies in incidence of disease and the way in which male and female musculoskeletal health can be affected. These differences reflect the action of different sex hormone levels, however further insights into the causes, manifestation and implications for treatment of the disparity between sexes are required if patient care is to be optimized.

Zuo et al. reported that the visceral adiposity index (VAI) is linked to a lower prevalence of osteoporosis, while others identify VAI as an independent predictor of trabecular bone loss. In addition, it is known that the influence of obesity and adiposity on osteoporosis manifests differently in men and women (1). In detail, the authors focused their attention on the relationship between Chinese VAI (CVAI) and marrow adiposity using a three-dimensional Fat Analysis & Calculation Technique sequence in postmenopausal women. The authors aimed to investigate the relationship between the Chinese visceral adiposity index (CVAI) and vertebral proton density fat fraction (PDFF). The study included 181 postmenopausal women including 53 with normal bone mineral density (BMD), 88 with osteopenia, and 40 with osteoporosis. A clear negative correlation between CVAI and PDFF after adjusting for age, time since menopause, waist circumference, body mass index, physical activity, and lipid profiles. The association with marrow PDFF was significant. The authors concluded that the moderate visceral fat accumulation may ameliorate skeletal integrity, whereas excessive visceral fat could possibly display negative effects.

Faber et al. reviewed Sex differences in the radiographic and symptomatic prevalence of knee and hip osteoarthritis, the most common forms of large joint osteoarthritis. Females seems to have a higher prevalence of knee osteoarthritis, while reports for the hip differ according to how the disease is defined. Symptomatically or clinically defined hip osteoarthritis is much more evident in females, whereas radiographically defined hip osteoarthritis is more frequent in males. Consequently, the sex differences underlying of large joint arthritis is matter of investigation. The basis for these differences may reflect a combination of hormonal, behavioral and biomechanical factors.

Zhao et al. studied the associations of different dietary patterns, bone mineral density, and fracture risk among elderly women: the China Osteoporosis Prevalence Study. China is rich of elderly people with high prevalence of osteoporosis and fractures. The study included 17,489 randomly sampled subjects aged ≥40 years. A diet rich in meat, vegetables, dairy, fruit, and egg was significantly linked to higher BMD at total hip, femoral neck, and lumbar spine. Interestingly, a diet rich in beverages and fried food was associated with a lower BMD at the femoral neck and lumbar spine. A diet rich in meat was associated with 34%–39% decreased risk of clinical fracture and vertebral fracture. Stronger associations were observed in women. Interestingly, postmenopausal women showed also a positive correlation between carnivorous and vegetarian diets and high BMD, and similarly between carnivorous diet and decreased fracture risk. Dairy and fruit also showed a beneficial effect on bone health. These findings suggested that nutritional recommendations for older people in China may be helpful in optimizing bone health, especially in postmenopausal women.

Rühling et al. evaluated sex differences and age-related changes in vertebral body volume and volumetric bone mineral density at the thoracolumbar spine using opportunistic quantitative computed tomography (QCT). The authors measured the age- and sex-related longitudinal changes in trabecular volumetric bone mineral density (vBMD) and vertebral body volume at the thoracolumbar spine in adults. The study included 168 adults. Thoracolumbar vBMD was higher in women compared with men before menopause, but were no longer evident after menopause, due to accelerated and more profound vBMD decline in women. Having excluded degenerative vertebrae, small increases were observed in vertebral body volume which were greater in males.

In conclusion, sex-related differences in bone disease and musculoskeletal health are frequently observed and likely reflect a combination of nutritional, hormonal, behavioral and biomechanical factors.
